# Protocol for designing, optimizing, and analyzing reverse supply chains using RELOG

**DOI:** 10.1016/j.xpro.2022.101913

**Published:** 2022-12-21

**Authors:** Chukwunwike O. Iloeje, Alinson Santos Xavier, John Atkins, Kyle Sun, Audrey Gallier

**Affiliations:** 1 Energy Systems and Infrastructure Analysis Division, Argonne National Laboratory, Lemont, IL 60439, USA; 2 Department of Mechanical Engineering, Arizona State University, Tempe, AZ 85281, USA; 3 Natural Power, Saratoga Springs, NY 12866, USA; 4 Khoury College of Computer Sciences, Northeastern University, Boston, MA 02115, USA

**Keywords:** Energy, Computer sciences, Environmental sciences

## Abstract

In this protocol, we describe the use of RELOG, an open-source, supply chain optimization package, for robust design and analysis of optimal reverse logistics and manufacturing networks. We detail installation steps and input data assembly, followed by problem modeling. We further detail how to run the RELOG optimization, visualize, and analyze the results. The implementation discussed here illustrates battery recycling; however, our package can analyze a wide variety of supply chains with multiple types of plants, products, and time periods.

For complete details on the use and execution of this protocol, please refer to Xavier and Iloeje (2020)^1^ and Iloeje et al. (2022).^2^

## Highlights

• RELOG is a broadly applicable supply chain optimization package
• Demonstrates implementation for modeling reverse supply chains
• Detailed steps for assembling input data and modeling problem
• Built-in and customizable data analysis and results visualization

## Abstract (editor highlights)

Publisher’s note: Undertaking any experimental protocol requires adherence to local institutional guidelines for laboratory safety and ethics.

## Graphical abstract

**Figure:**
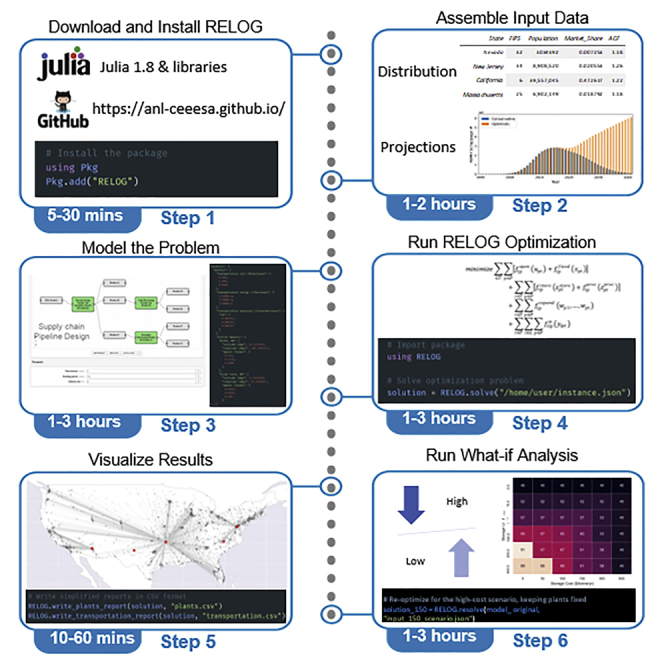

## Before you begin

1. Scope.

This protocol describes the application of RELOG^1^ for designing and optimizing reverse supply chains, i.e., supply chain pipelines that start from a single product and generate multiple material streams in one or more stages. The specific application described here is the techno-economic analysis of critical material recycling from spent hybrid electric vehicle (HEV) batteries. The overall steps of the protocol, however, also apply to any reverse supply chain analysis, such as lithium-ion battery recycling, electronics recycling, or low carbon feedstock (e.g., biomass) supply for chemicals manufacturing. While the geographical region here is the United States, it can be used for any region, if the geographic information data (latitudes and longitudes) are available. The advantage of employing RELOG for such analysis is that it provides a robust, system-level techno-economic assessment of recycling, extending the analysis boundary beyond a single facility, and accounting for stocks, time-resolved availability and the logistics of collecting and transporting the primary feedstock, in this case, the spent HEV batteries. The costs and material flow outputs from the RELOG model can inform other downstream analysis, such as life cycle assessment.

2. Structure.

RELOG is an open-source package for design and analysis of optimal reverse logistics and manufacturing supply chain networks. It supports customized reverse logistics pipelines with multiple types of plants, multiple types of products, and multiple periods. It also allows for storage and plant capacity expansion, and tracks costs, emissions and energy use. Key supply chain decisions include recycling/processing plant location and sizing, customer allocation and optimal material flow. The package is written in the Julia programming language and uses Mixed-Integer Linear Optimization (MILP) to find optimal decisions. Further details on the MILP formulation and solution methods have been included in the software documentation.^1^

3. Insights.

RELOG can provide insights for a wide range of analysis categories. For facility location, sizing and upgrade assessments, RELOG can answer questions such as (1) how many facilities should be built, where and when? (2) Which customers should be served by each facility? (3) What type of facility, and how large? (4) Which facilities should be upgraded? The package can also be used for marginal cost analysis to provide additional insights on economic feasibility, addressing questions such as (1) what is the cost to process an extra unit of material given existing network? (2) Would a profit-maximizing firm be interested in pursuing this enterprise? (3) If not, what types of incentive structures would be necessary? RELOG can also be used for what if analysis to assess sensitivity to changes in material demand, supply or cost parameters.

4. Hardware and Software Requirements.

RELOG can run on all operating systems supported by the Julia programming language, including Windows, macOS and Linux. Hardware requirements are heavily dependent on the size and complexity of the reverse logistics pipeline being optimized, but a modern quad-core processor and at least 8 GB of RAM are generally recommended. By default, RELOG internally uses open-source MILP solvers (Cbc^3^ and HiGHS^4^) to find optimal solutions. Better performance can typically be achieved, on the same hardware, by using a state-of-the-art commercial MILP solver, such as Gurobi.^5^

5. User Interface.

**Figure 1:**
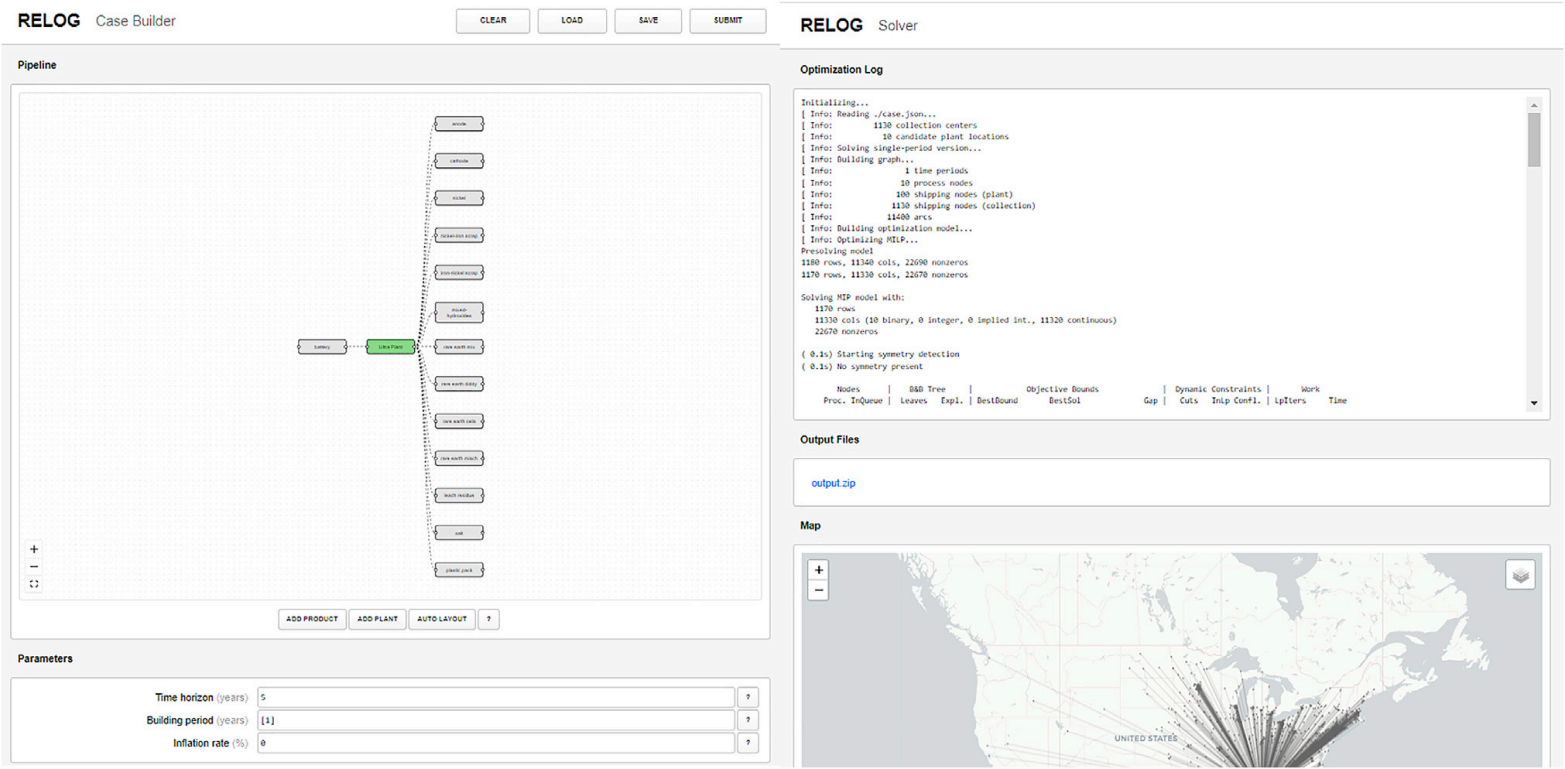
Screenshots of the RELOG web interface Left: Case builder, which allows the user to quickly prototype test cases. Right: Solver page, which displays optimization results and a graphical visualization of the solution.

RELOG currently provides two user interfaces: (1) a publicly accessible web interface, which allows for quick prototyping and solution of simple test cases, without requiring the installation of any additional software, or any programming skills from the user (see Figure 1); and (2) a Julia programming interface, which allows for the solution of more complex test cases, and which provides more customization options, such as the usage of a commercial MILP solver. In the steps below, we will use both interfaces to conduct our analysis.

6. Interactive Jupyter Notebooks.

We have provided a set of Jupyter Notebooks,^6^ written in Julia^7^ and Python,^8^ which reproduce the implementation described in the “step-by-step method details” section. These can be accessed using the DOI provided in the key resources table.

## Key resources table

**Table:** 

REAGENT or RESOURCE	SOURCE	IDENTIFIER
**Deposited data**		

All input and output data (JSON and CSV files) for the case studies	This paper	ZENODO: https://doi.org/10.5281/zenodo.7093835

**Software and algorithms**		

RELOG	Argonne National Laboratory https://anl-ceeesa.github.io/RELOG	ZENODO: https://doi.org/10.5281/zenodo.5131239
Interactive Python and Julia notebooks illustrating method details	This paper	ZENODO: https://doi.org/10.5281/zenodo.7093835

## Step-by-step method details

The following content describes in detail the steps to conducting reverse logistics network design and optimization using RELOG, illustrated for the HEV battery recycling supply chain analysis.

### Step 1: Download and install RELOG and dependencies

**Timing: 5–30 min**

This section describes the steps for downloading and installing the RELOG package. It is a one-time activity, and the first of the four critical stages this protocol.

#### Using the Julia programming interface

1. The steps below assume that the following dependencies have been correctly installed and configured:
  a. Julia Programming Language, version 1.8.^7^
  b. Python Programming Language,^8^ version 3.9.
  c. Jupyter Notebook,^6^ version 3.4.
  d. Julia kernel for Jupyter,^9^ version 1.23.

We recommend installing Python and Jupyter through the Anaconda Distribution.^10^

2. To install RELOG on your local machine, launch the Julia console, then run:

using Pkg

Pkg.add(name="RELOG", version=“0.5”)

3. After the package has been installed, please run the RELOG test suite, as shown below, to make sure that the package has been correctly installed:

Pkg.test(“RELOG")

***Note:*** An interactive Julia Notebook (*Download and Install RELOG.ipynb*) is available for the steps in this section.

#### Using the web interface

4. No downloads are required for using the RELOG Case Builder. Simply navigate to the website URL^11^ on your preferred browser.

### Step 2: Assemble input data

**Timing: 1–2 h**

**Figure 2:**
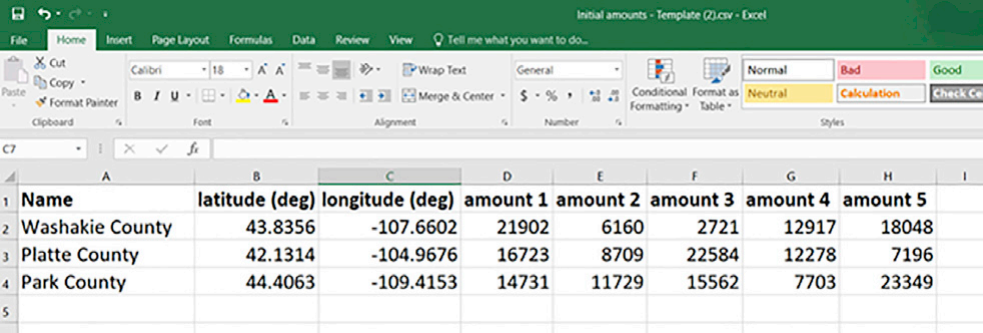
Screenshot of Excel/CSV template for initial material amounts Each “amount” column represents a time-period (e.g., year).

**Figure 3:**
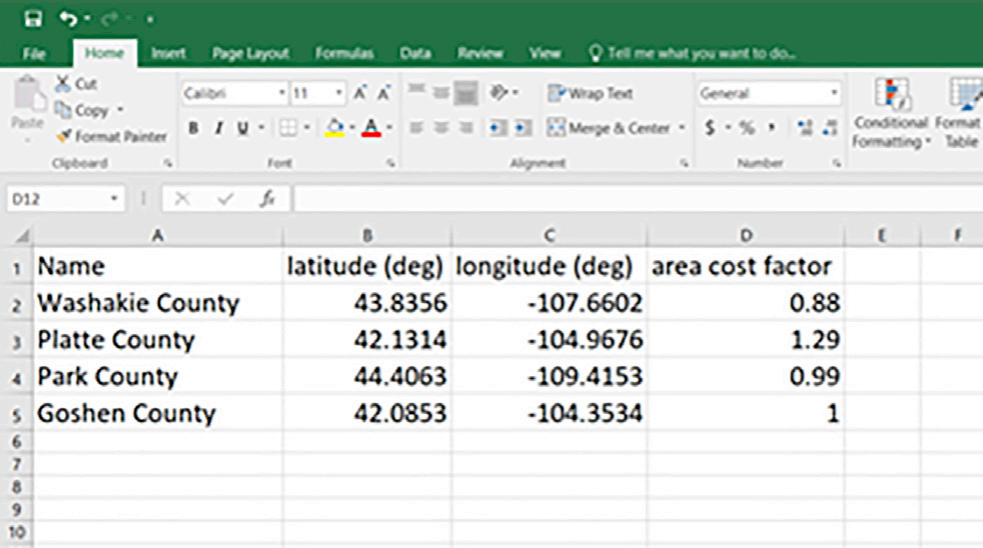
Screenshot of Excel/CSV template for candidate locations Additional information such as ‘regional cost factors’ can also be provided.

This section outlines the key data requirements for the supply chain pipeline for a typical case study and is the second of the four critical stages of this protocol. RELOG requires data to specify the products and plants described in the pipeline. For products, it accepts geospatial data for the distribution of primary feed, then other information such as initial amounts (at each location), and transport data (cost, energy and emissions). For plants, it accepts geospatial distribution of candidate locations for each type of plant, and other plant specific data such as material balance (input and output ratios), capacities (minimum and maximum), economics (opening, fixed and variable operating and disposal costs), specific energy use and emissions. Geospatial data can be obtained from geographic information system databases and resource repositories,^12^^,^^13^ and can be provided in .csv format (see screenshots in Figures 2 and 3). Other data should come from appropriate sources relevant to the specific case study.

#### Using the web interface

5. Create Excel/CSV file and populate with initial amounts and distribution.
  a. Download the template for initial product amount and distribution (Figure 2) from the web interface.
  b. Populate it with spatial and temporal distribution data for the initial product.
6. Create Excel/CSV file and populate with candidate locations.
  a. Download the template for candidate plant locations (Figure 3).
  b. Populate it with location data.
7. Collate other material flow, energy, emissions and economic data associated with each product and plant type. See “Model the Problem” section for details.

#### Using the Julia Programming Interface

In general, we recommend using the web interface for assembling the input data (this section) and for modeling the problem (next section). If desired, however, it is also possible to skip the web interface and directly create a RELOG input file in any programming language. RELOG accepts as input a JSON file with three sections, describing general optimization parameters, products and plants. The format of this file is described in detail in the RELOG documentation.^1^

***Note:*** We have included a sample initial amounts data file (“*InitialAmounts.csv*”) and a sample candidate locations data file (“*CandidateLocations.csv*”) with the accompanying input data files (see key resources table). The steps described above equally apply for when using the programming interface as well as when using the web interface.

***Note:*** In the candidate locations CSV, you may also include additional data such as “area cost factor” to account for differences in construction costs across U.S. regions.^14^

### Step 3: Model the problem

**Timing: 1–3 h**

This section describes how to model the problem before submitting it to RELOG for optimization. It is the third of the four critical stages of this protocol. In RELOG, reverse logistics pipelines are described by two main model components: (1) *products*; and (2) *processing plants*. A product is any material that needs to be recycled, any intermediary product produced during the recycling process, or any product recovered at the end of the process while a plant is a facility that converts one type of product to another. In this case study, we had 14 types of products: (1) NiMH batteries; (2) cathode; (3) anode; (4) nickel (Ni) metal; (5) nickel-iron (Ni-Fe) scrap; (6) iron-nickel (Fe-Ni) scrap; (7) mixed hydroxides; (8) mixed REE chlorides (Rare-Earth Mix); (9) didymium (Nd-Pr) alloy; (10) cerium-lanthanum (Ce-La) alloy; (11) mischmetal (misch) alloy; (12) leach residue; (13) salt; and (14) plastic pack. NiMH batteries are the primary source materials. Cathode, anode and mixed REE chlorides are intermediate products, whereas the remaining products are final products. Since we enforced co-location, we combined these into a single plant that takes batteries as input, and final products as outputs. The following steps describe how to specify products and plants, and provide the information to RELOG as a JSON file.

#### Using the web interface

**Figure 4:**
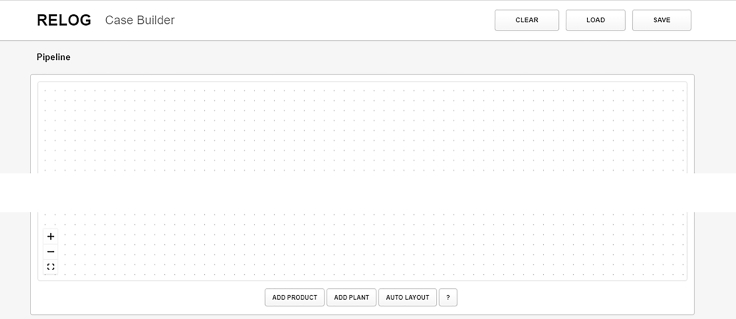
Screenshot of the pipeline design canvas on the Case Builder Web Interface
  b. Use the “*add product*” and “*add plant*” buttons to drop and name product streams and plant nodes onto the canvas.
    i. Connect the product and plant objects by clicking and dragging from an upstream to a downstream port until you complete the pipeline (Figure 5).

8. Design the pipeline:
  a. Navigate to the **RELOG Case Builder**^11^ on your preferred browser. The page opens to a blank canvas illustrated in Figure 4.

**Figure 5:**
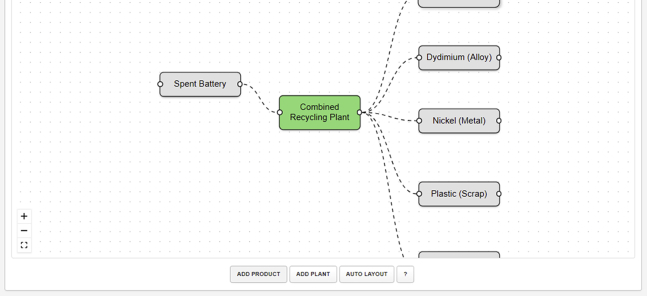
Screenshot of example Pipeline for Battery Recycling

***Note:*** Ports are the small circles on either side of each product or plant object.

**Figure 6:**
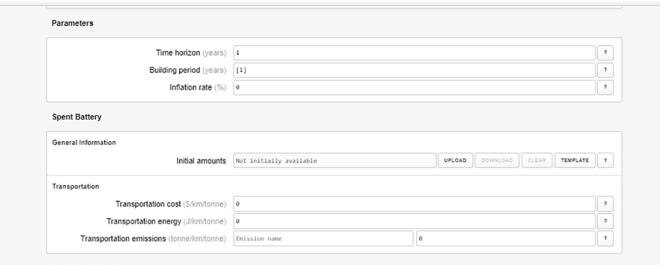
Screenshot of form elements for specifying simulation parameters and products

**Figure 7:**
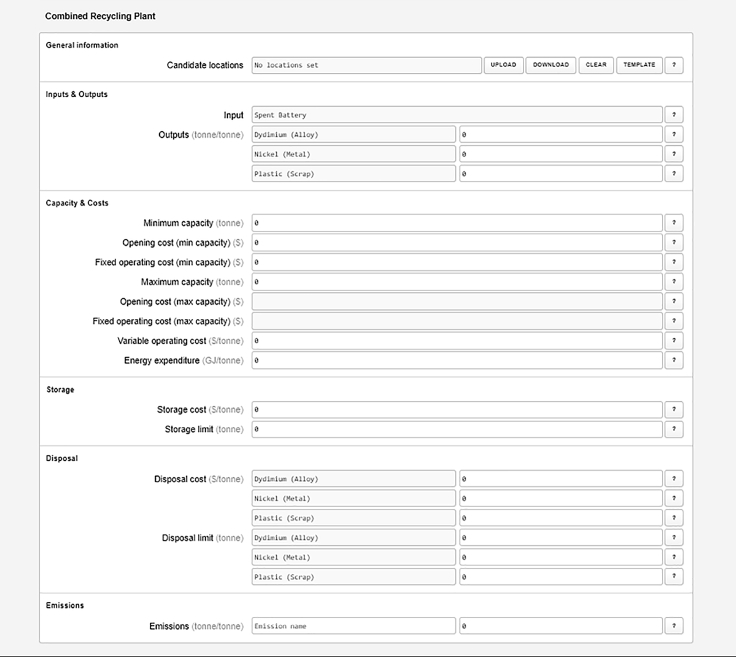
Screenshot of form elements for specifying plants

9. Specify the model:
  a. Once you complete the pipeline, according to the previous step, the Case Builder will automatically generate data entry forms for each pipeline element. Scroll down the page to view them.
  b. Enter the overall model parameters.
    i. Parameters include *Time horizon* – number of time periods to be considered in the simulation (e.g., years); *Building period*, which specifies period during which new plants can be opened, and *inflation rate* (Figure 6).
      ***Note:*** A single time period can be specified if there is no interest in exploring temporal evolution within, say, a multi-year period.
  c. Specify the products. The required information includes transportation cost, energy and emissions (Figure 6).
    i. For primary products, use the “*upload*” button to specify the initial amounts data.
    ii. Add the corresponding data for all the other “product” form elements.
  d. Specify the plants: The required data categories include general information (candidate plant locations), inputs and outputs, plant capacities and costs, storage, disposal and emissions (Figure 7).
    i. Use the “*upload*” button to load the candidate location data file.
10. Generate the input JSON file:
  a. Once model specification is complete, click the “save” button on the top right corner of the page (Figure 4) to generate the JSON input file.
  b. Move the file to the appropriate project folder if your browser download directory is a different location.

#### Using the Julia programming interface

We recommend using the web interface for modeling the problem. For directly generating the JSON file without using the RELOG Case Builder, see the RELOG documentation.^1^

***Note:*** All the relevant data files for both conservative and optimistic scenarios - fully specified JSON inputs, initial amounts and candidate locations - considered in this HEV battery recycling case study have been made available (see key resources table). Instead of building from scratch, the user can load the JSON input files onto the Case Builder Web Interface using the “Load” button at the top of the browser page. This will load the pipeline and associated data. The user can then modify the pipeline and the associated data as needed.

### Step 4: Run RELOG optimization

**Timing: 1–3 h**

This section describes job submission to solve the supply chain problem in RELOG. This is the fourth and final critical stage of this protocol. After creating a JSON file describing the reverse manufacturing process and the input data, the following steps illustrate how to use the package to find the optimal set of decisions.

#### Using the Julia programming interface

11. Submit RELOG input file for optimization.
  a. The code snippet below illustrates how to submit the optimization problem to RELOG. The path *“input/smallproblem/InputPipelineData.json”* points to JSON file generated in step 10. Replace with correct file path.

*# Import package*

using RELOG

*# Solve optimization problem*

solution = RELOG.solve("input/smallproblem/InputPipelineData.json")

12. Write the solution to CSV files.

*# Write CSV report showing plant costs, capacities, energy expenditure and*

*# utilization factors*

RELOG.write_plants_report(solution, "output/smallproblem/plants.csv")

*# Write CSV report showing amount of product sent from initial locations to plants,*

*# and from one plant to another. Includes the distance between each pair of*

*# locations, amount-distance shipped, transportation costs and energy expenditure*

RELOG.write_transportation_report(solution, "output/smallproblem/transportation.csv")

*# Write CSV report showing primary product amounts, locations and marginal costs*

RELOG.write_products_report(solution, "output/smallproblem/products.csv");

#### Using the web interface

It is also possible to run the optimization problem on RELOG from the Case Builder interface, using the “SUBMIT” button on the top right side of the web browser page. However, this was set up for simple evaluation purposes, so it uses an open-source mixed-integer linear programming solver and places a strict limit on memory and optimization time. Therefore, it is not ideal for solving large problems, but can be used for quick evaluation and visualization of smaller-sized problems. In general, problem difficulty scales with the number of time periods, initial product locations, and candidate facility locations.

**Note 1**: An interactive python notebook (“*Submit Optimization Problem.ipynb*”) is available for the steps in this section. For a detailed list of report options and content descriptions, please see the RELOG documentation.^1^ We have included appropriate input files for a small-sized problem with the accompanying input data files (see key resources table). These files can be loaded and optimized directly from the Case Builder, for evaluation purposes.

**Note 2:** As previously mentioned, RELOG by default uses open-source mixed-integer linear programming solvers to find optimal solutions. For larger-scale test cases, a commercial solver such as Gurobi, CPLEX or XPRESS is recommended. The following snippet shows how to switch to Gurobi:

using RELOG, Gurobi

RELOG.solve("instance.json", optimizer=Gurobi.Optimizer)

### Step 5: Visualize RELOG results

**Timing: 10–60 min**

This section discusses how to visualize simulation results, which is an important, though non-critical stage of the protocol. RELOG generates a number of simplified reports in tabular data format (CSV) for easy processing with spreadsheet software (e.g., Microsoft Excel) or by data analysis libraries (e.g., pandas^15^). These can also be used to generate visualizations in spreadsheet software and other graphical analysis libraries. We illustrate sample charts produced using Python visualization libraries. Sample report files are also included with the accompanying output data files (see key resources table).

**Figure 8:**
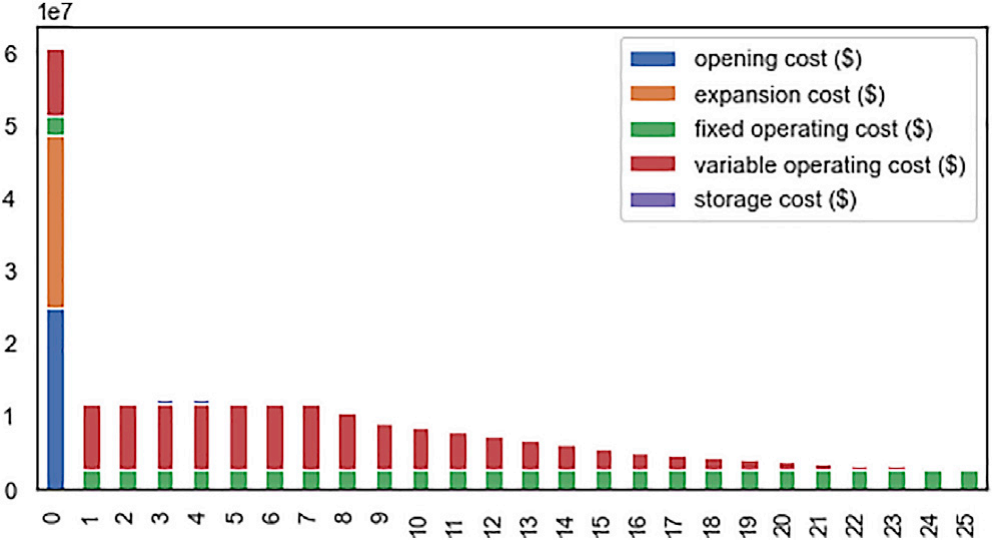
Bar plot with annual breakdown of plant costs

***Note:*** Make sure your python installation includes pandas,^15^ matplotlib^16^ and seaborn.^17^

13. Visualize plant data using Python:
  a. Run the Python code snippet below to create bar plots showing total plant costs by year, grouped by plant type (Figure 8).

import matplotlib.pyplot as plt

import pandas as pd

import seaborn as sns

sns.set_style("white")

columns = [

**Figure 9:**
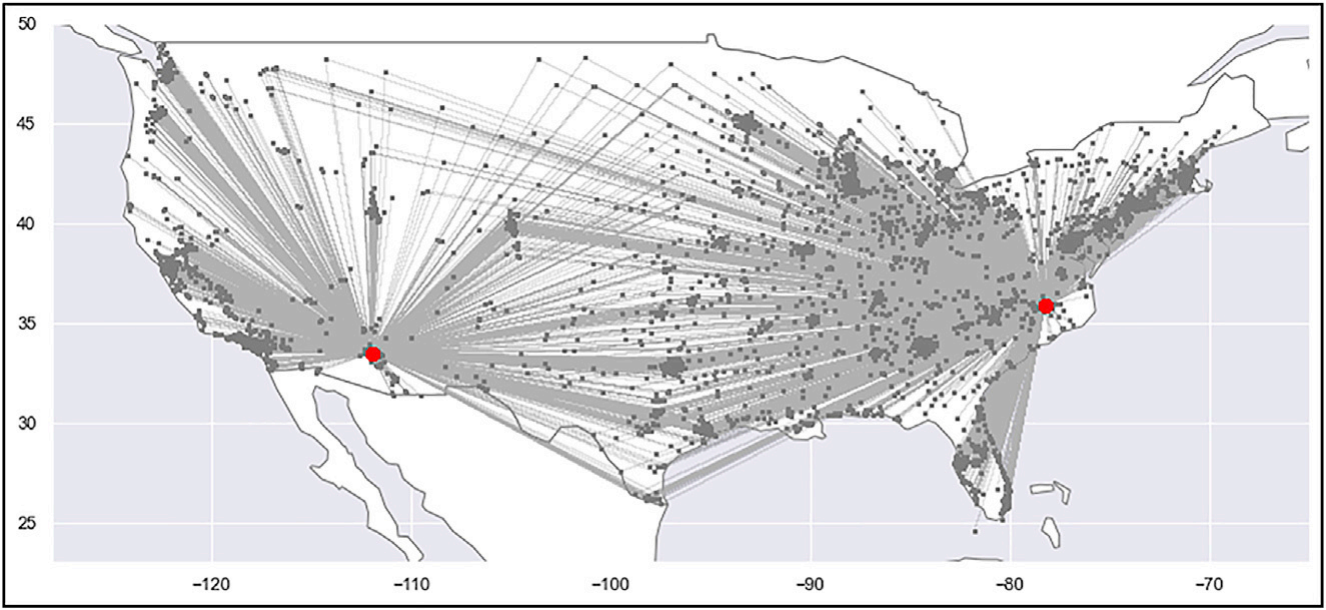
Map showing cost-optimized plant (facility) locations and transportation lines from RELOG simulation for the case study example Transportation routes between collection centers and recycling facilities here are shown as straight-line approximations. The number, size and location of facilities depend on the trade-off between economies of scale, and proximity to collection centers (transport cost), and in this case, favors centralized plants.

***Note:*** In addition to the libraries previously mentioned, the snippet below also requires geopandas^18^ and shapely.^19^

 "opening cost ($)",

 "expansion cost ($)",

 "fixed operating cost ($)",

 "variable operating cost ($)",

 "storage cost ($)",

]

data = pd.read_csv("output/conservative/plants_report.csv")

df = data.groupby(["plant type", "year"]).sum().reset_index()

df[columns].plot(kind="bar", stacked=True, figsize=(8, 4))

plt.savefig("figures/conservative/plant_costs_breakdown.pdf", dpi=300);

14. Visualize transportation data using Python:
  a. Run the Python code snippet below to generate a map of transportation lines connecting collection centers with recycling facilities (Figure 9).

import geopandas as gp

import matplotlib.pyplot as plt

import pandas as pd

from matplotlib import collections

from shapely.geometry import LineString, Point

*# Plot base map*

world = gp.read_file(gp.datasets.get_path("naturalearth_lowres"))

ax = world.plot(color="white", edgecolor="0.5", figsize=(14, 7))

ax.set_ylim([23, 50])

ax.set_xlim([-128, -65])

*# Draw transportation lines*

data = pd.read_csv("output/conservative/transportation_report.csv")

lines = [

 [

  (

   row["source longitude (deg)"],

   row["source latitude (deg)"],

  ),

  (

   row["destination longitude (deg)"],

   row["destination latitude (deg)"],

  ),

 ]

 for (index, row) in data.iterrows()

]

ax.add_collection(

 collections.LineCollection(

  lines,

  linewidths=0.01,

  zorder=1,

  alpha=0.5,

  color="0.7",

 )

)

*# Draw source points*

points = gp.points_from_xy(

 data["source longitude (deg)"],

 data["source latitude (deg)"],

)

gp.GeoDataFrame(data, geometry=points).plot(ax=ax, color="0.5", markersize=1)

*# Draw destination points*

points = gp.points_from_xy(

 data["destination longitude (deg)"],

 data["destination latitude (deg)"],

)

gp.GeoDataFrame(data, geometry=points).plot(ax=ax, color="red", markersize=50)

plt.savefig("figures/conservative/recycling_logistics.pdf", dpi=300);

15. Visualize plant locations and transportation lines in the web interface:
  a. If the problem is submitted directly via the RELOG Case Builder, the web page automatically loads a map which displays the chosen plant locations as well as the transportation lines between plants and collection centers, as illustrated in Figure 9.
  b. For generating other charts and bar plots, you may use another tool such as Python, as described in the documentation.^1^
  c. Future versions of the Case Builder web interface will include options for generating custom charts and plots.

***Note:*** An interactive python notebook (“*Visualize RELOG Results.ipynb*”) is available for the steps in this section.

### Step 6: Run what-if analysis

**Timing: 1–3 h**

This final method details section presents additional analysis options using RELOG. It is not critical to using this package but is potentially useful for drawing additional insights from analysis. In general, RELOG decides when and where to build plants based on a deterministic optimization problem that minimizes costs for the specific scenario provided by the user. In practical situations, some of the input parameters such as costs, demands and emissions may be uncertain or not known with confidence. In this situation, RELOG can evaluate how well does the optimized facility location plan produced by RELOG works if any of these parameters turn out to be different, using the “resolve” method. Parameters that can be updated include products (transportation costs, energy, emissions and initial amounts - latitude, longitude and amount) and plants (energy, emissions, opening, fixed, variable and product storage costs, and location - Latitude and longitude).

#### Using programming interface

16. Generate What-if analysis scenarios.
  a. Run the Python code snippet below to generate what-if analysis scenarios via a python programming interface. The code generates scenarios with varying combinations of battery *storage cost* and *storage limit (See* Figure 7 *and Note 2).*
    import json
    max_capacity = 5628
    with open("input/conservative/InputPipelineData.json") as infile:
     case = json.load(infile)
     t = case["parameters"]["time horizon (years)"]
     for cap in [0.0, 0.25, 0.5, 1, 2, 4]:
      for cost in [0.0, 50, 100, 150, 300, 600]:
       for plant in ["Ultra Plant"]:
        for loc in case["plants"][plant]["locations"].values():
         loc["storage"]["cost ($/tonne)"] = [cost for _ in range(t)]
         loc["storage"]["limit (tonne)"] = cap ∗ max_capacity
       with open(
        f"input/whatif/storage_ultra_cap_{cap}_cost_{cost}.json",
        "w",
       ) as outfile:
        json.dump(case, outfile, indent=2)
  b. Run the code snippet to submit the original optimization and the what-if scenarios for RELOG optimization via a Julia programming interface.
    using RELOG
    using Glob
    *# Solve reference case*
    _, model = RELOG.solve("input/conservative/InputPipelineData.json", return_model=true);
    *# For each what-if scenario...*
    for filename in glob("input/whatif-conservative/∗.json")
     *# Solve scenario*
     solution = RELOG.resolve(model, filename)
     *# Write CSV reports*
     prefix = joinpath(
      "output",
      "whatif-conservative",
      replace(basename(filename), ".json" => ""),
     )
     RELOG.write_plants_report(solution, "$(prefix)_plants.csv")
     RELOG.write_products_report(solution, "$(prefix)_products.csv")
     RELOG.write_plant_outputs_report(solution, "$(prefix)_plant_outputs.csv")
     RELOG.write_plant_emissions_report(solution, "$(prefix)_plant_emissions.csv")
     RELOG.write_transportation_report(solution, "$(prefix)_tr.csv")
     RELOG.write_transportation_emissions_report(solution, "$(prefix)_tr_emissions.csv")
    end

#### Using case builder web interface

The what-if analysis scenarios can also be easily generated via the Case Builder Web Interface.

17. Load the original input file (“*InputPipelineData.json*”), change the parameter(s) to be varied for a given scenario, save the updated file and rename the saved file appropriately.
18. Repeat this for each scenario.
19. Evaluate as described in 16a.

***Note:*** Interactive python notebooks are available for step 16a (“*Generate What-if Scenarios.ipynb*”) and step 16b (“*Submit What-if Scenarios.ipynb*”) in this section. An additional interactive notebook has also been included to visualize outcomes from the “what-if” scenario analysis (“*Visualize What-if Scenarios.ipynb*”).

***Note:*** Note that the “max_capacity” parameter simply refers to the **maximum capacity** for the recycling plant (See Figure 7). In this case, we are running scenarios where we determine the **storage limit** to be 0–4 times the maximum annual plant capacity (e.g., cap = 0.25 will replace “storage limit” value in Figure 7 with **0.25∗max_capacity**), and the **storage cost** to vary from $0.0 to $600/tonne of capacity used.

## Expected outcomes

**Figure 10:**
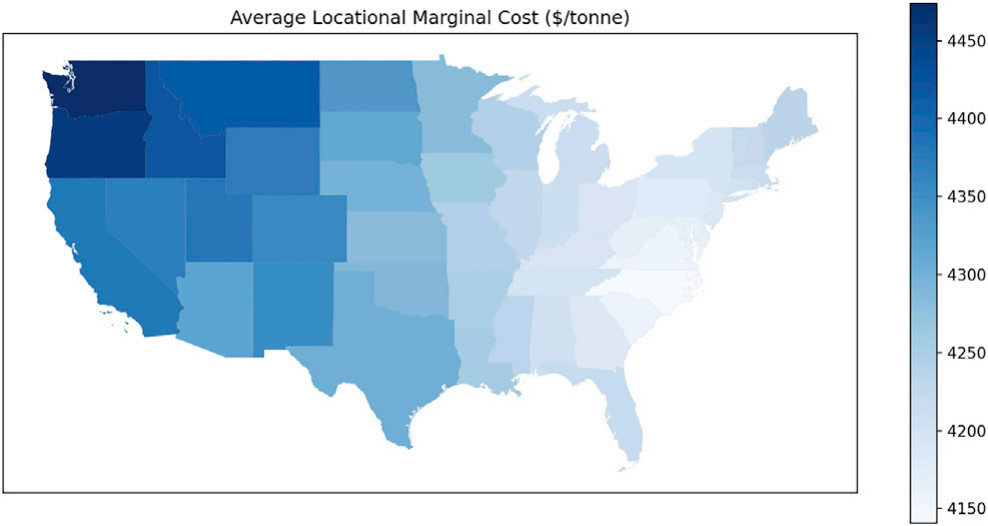
Locational marginal costs for case study example on NiMh battery recycling in the U.S. The locational marginal costs represent the cost of recycling additional tonne of battery from a given location In this example, it provides an economic metric for guiding decisions of profit maximizing recycling plants since they would decide to recycle additional material only if the expected revenue exceeds the marginal cost.

**Figure 11:**
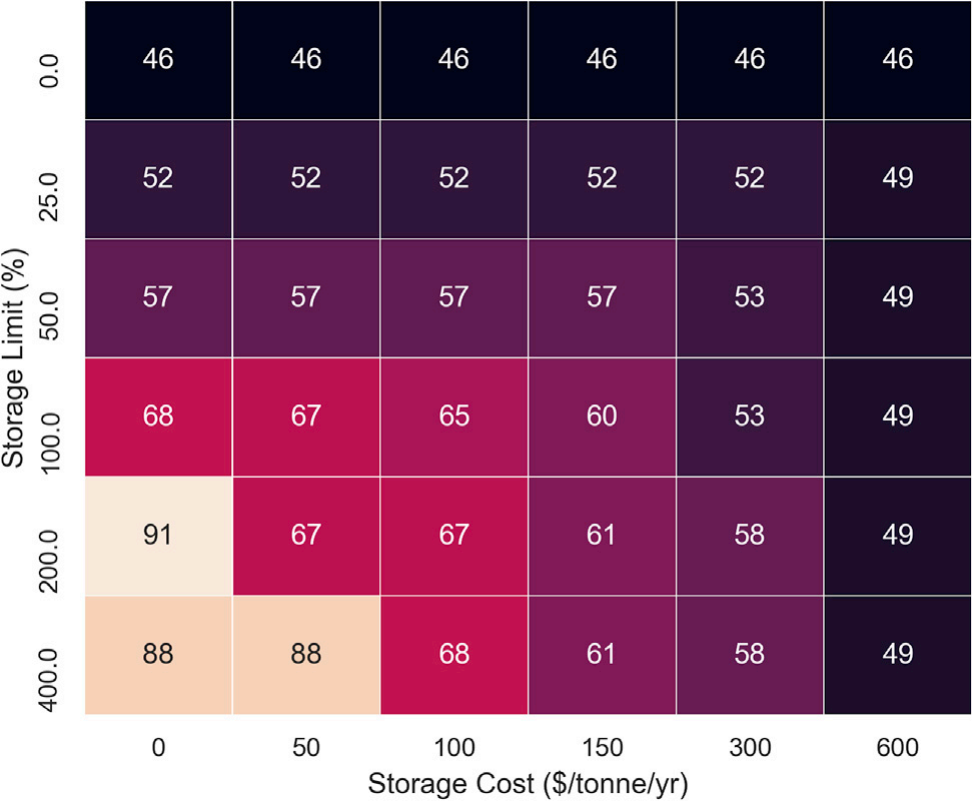
Heat map showing the variation of recycling facility utilization (%) with changes in storage cost ($/tonne/yr) and storage limit (% of total capacity) This illustrates an outcome from the “What-if” analysis, which investigates the impact of adjusting selected levers – in this case, storage cost, or storage capacity – on a target supply chain metric – in this case, the degree of utilization of the recycling plants. These results suggest that a supply chain network design that favors larger storage facilities, and possibly lower costs due to economies of scale – improves overall utilization.

The preceding section details the different types of analysis outcomes from using RELOG to design an optimal reverse logistics infrastructure for critical material recycling. The main outputs range from material flows, transport and processing costs (with cost breakdowns) to energy and emissions and plant utilization factors. RELOG outcomes can be used as input for further analysis to provide insights on other relevant impacts. For instance, RELOG predicts marginal cost values, which can be visualized (using any GIS software or code library) and used to understand how the cost of processing an additional ton of battery varies with location (see Figure 10). Such information can inform the design of incentives to promote recycling. What-if analysis can also show how changes in some input parameters affect predictions. For instance, Figure 11 illustrates the variation of recycling facility utilization (%) with storage cost and limit. RELOG outputs can also serve as inputs to life cycle analysis to understand the relative contributions of different components of the revers supply chain to life cycle environmental impacts.

## Limitations

In the current version of RELOG, the following underlying assumptions can affect the validity of simulation results: (1) Each plant can only be opened exactly once. After open, the plant remains open until the end of the simulation. (2) Plants can be expanded at any time, even long after they are open. (3) All material available at the beginning of a time period must be entirely processed by the end of that time period. It is not possible to store unprocessed materials from one time period to the next. (4) Up to two plant sizes are currently supported. It does not support a nonlinear scaling for cost. (5) Variable operating costs must be the same for all plant sizes. This is not always the case in actual plants. (6) Transportation approximates straight-line distances between points, which underestimates overall transport costs by 10%–15%. Correction factors for approximating actual road-traced distances travelled can be used to address this.

## Troubleshooting

### Problem 1

**Figure 12:**
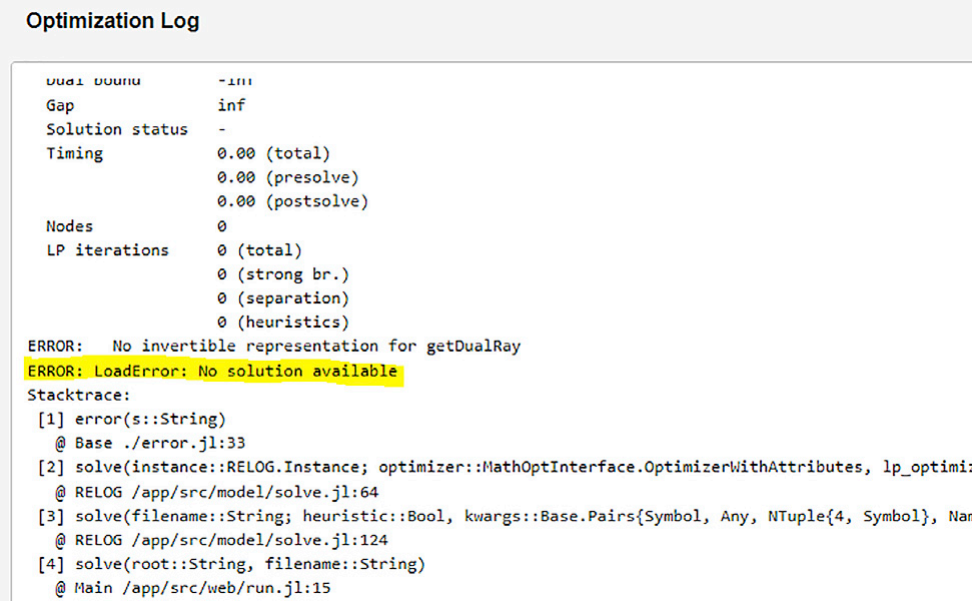
Screenshot of an infeasible problem

The problem is infeasible; RELOG returns a “no solution available” exception as illustrated in Figure 12 below (related to step 4).

### Potential solution

The most common factor that leads to solution infeasibility is the failure of mass balance. RELOG is formulated such that all the initial product (feedstock) amount must either be immediately processed, stored for future processing or disposed of. Therefore, the total maximum installed capacity of the plants/facilities must be greater than or equal to the total initial amount of materials available, less the amount disposed prior to processing. A simple back-of-the-envelope check will reveal this mismatch.

### Problem 2

**Figure 13:**
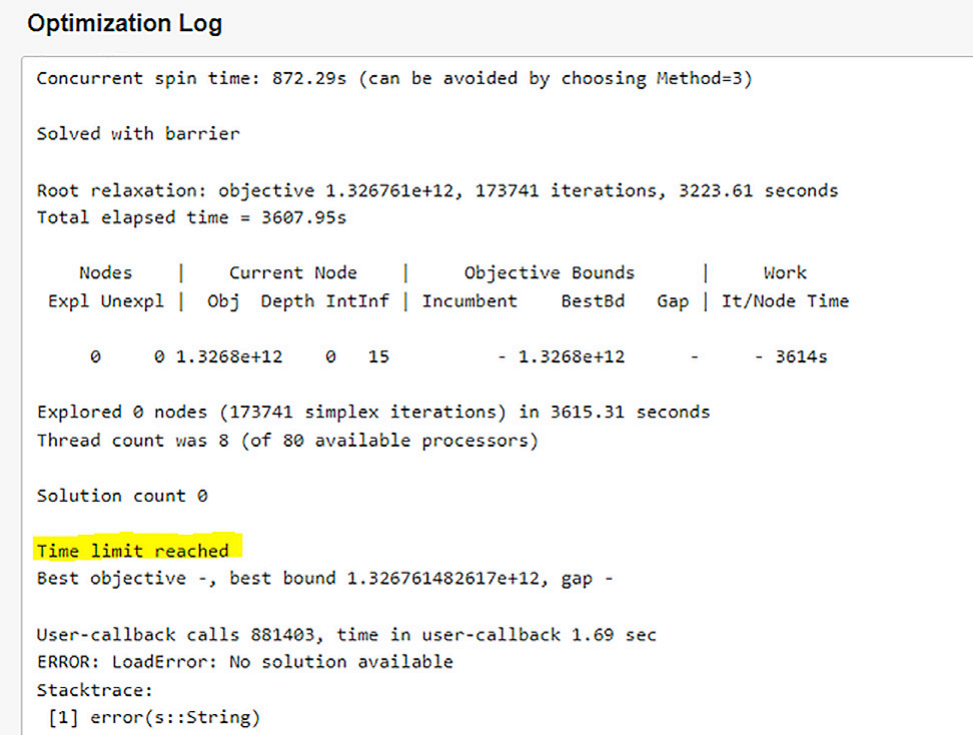
Screenshot of “timed out” exception

The problem is too large to solve; the optimization process either stalls or RELOG returns a “time limit reached” exception, as illustrated in Figure 13 below (related to steps 3 and 4).

### Potential solution

This problem may arise when the user tries to solve a large problem on a machine that lacks the computational resources to handle the problem, such as on the public server via the RELOG case builder interface (the public interface runs on a server with restrictions on memory and solution time, so is designed only to test relatively small problems). One alternative to resolve the issue is to optimize the case on a more powerful computer; this protocol describes how to install RELOG on a local computer or in-house server, which can handle much larger problems. Alternatively, another solution is to make the case smaller, by removing or combining collection centers and candidate plant locations.

### Problem 3

**Figure 14:**
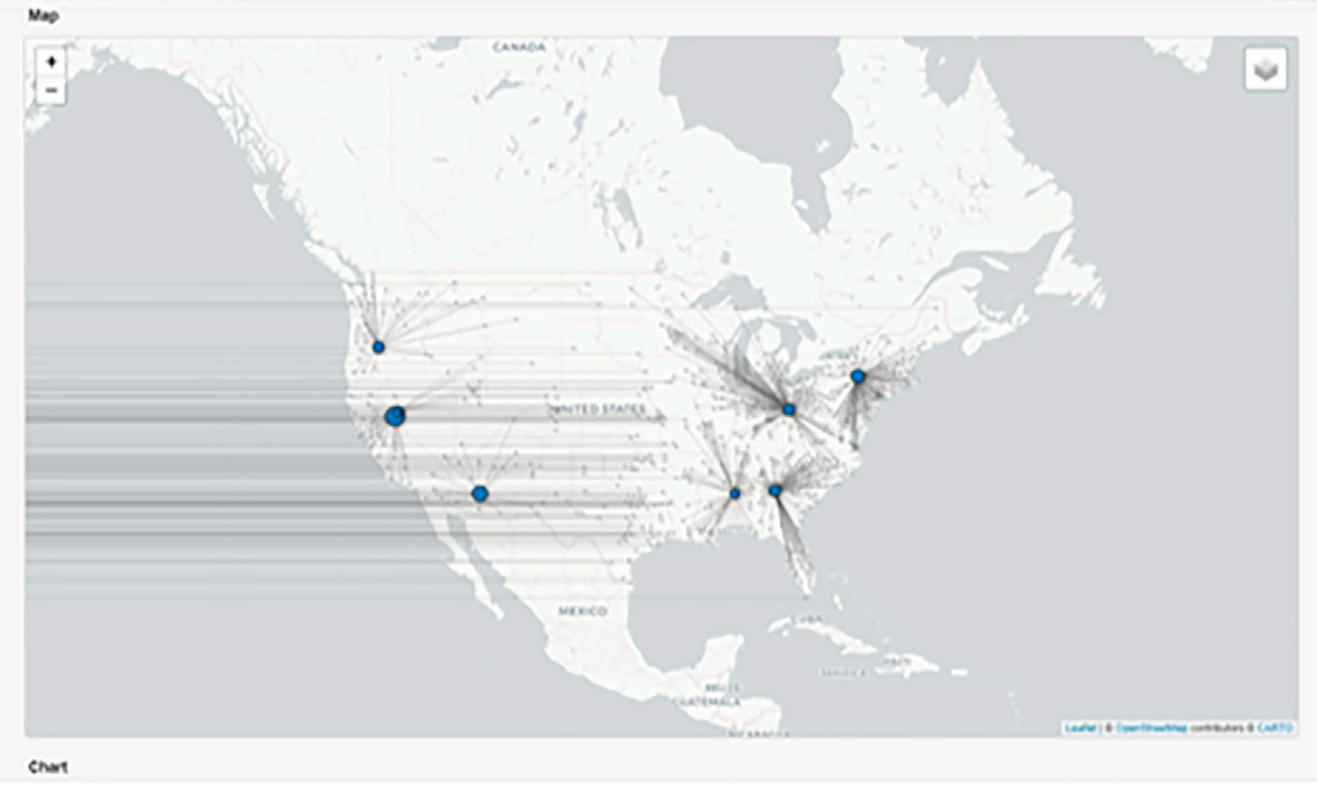
Screenshot of “unusual location” outcomes, where some plants are located outside the region of interest

RELOG outputs an unusual network with facilities located outside the region of interest, as illustrated in Figure 14 (related to step 4).

### Potential solution

This situation arises if there is some error with generating the location latitude and longitudes. A quick check to ensure realistic location coordinates would prevent the problem.

### Problem 4

The upload button in the RELOG case builder does not work (related to step 3).

### Potential solution

Check the format of the file to be sure it matches the destination field. For instance, ensure you are not trying to upload a JSON file when a CSV (e.g., candidate location) file is expected. Also, check to ensure that the column headers in the “initial amount” and “candidate location” files match the expected format. We recommend downloading the templates available in the case builder and using them as guide.

### Problem 5

**Figure 15:**
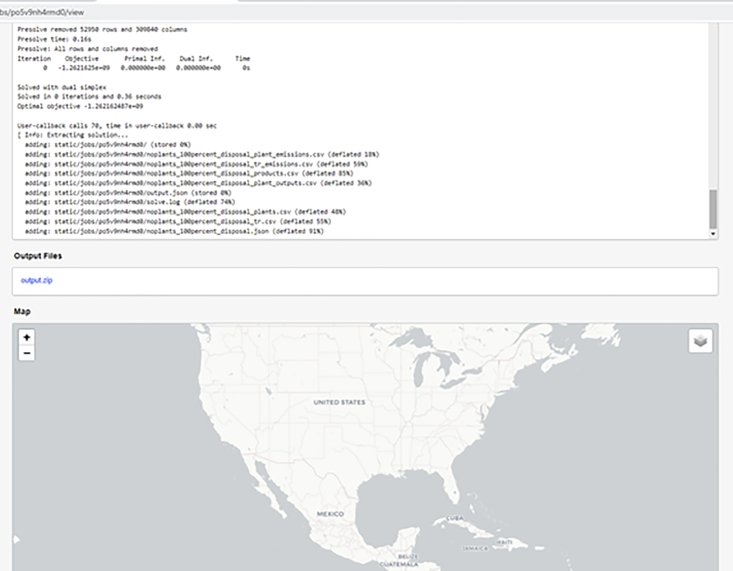
Screenshot showing “no plants/facilities opened” at end of simulation

RELOG solution does not open any plants, and some generated CSV files are empty. Such a result is illustrated in Figure 15 below (related to step 4).

### Potential solution

Check the input specifications for “disposal” of primary feedstock. By default, RELOG minimizes the overall cost of the logistics infrastructure network. If feedstock (or any product) disposal is unrestricted (100%) and cheap (e.g., $0.0), the solution will favor complete disposal, leading to the situation highlighted in Figure 15. If this is not intended, then specify appropriate costs or limits on disposal.

### Problem 6

Facility location and sizing results appear counter-intuitive. For instance, all plants are opened at the largest capacity with low utilization (related to steps 5 and 6).

### Potential solution

This problem often arises when the capacities are switched, i.e., larger capacity data is entered for the smaller plant and vice versa. A thorough check to ensure correct data entry will address this problem.

## Resource availability

### Lead contact

Further information and requests for resources and reagents should be directed to and will be fulfilled by the lead contact, Chukwunwike Iloeje (ciloeje@anl.gov).

### Materials availability

This protocol did not generate new materials.

## Data Availability

Data: Input files relevant to the case study (in JSON format) as well as the results data (in CSV format) have been deposited at Zenodo (https://doi.org/10.5281/zenodo.7093835) and are publicly available as of the date of publication. Code: The source code for the reverse logistics optimization model has been deposited at Zenodo (https://doi.org/10.5281/zenodo.5131239) and is publicly available as of the date of publication. Any additional information required to reanalyze the data reported in this paper is available from the lead contact upon request.
